# Supplementary material for: Compensatory Effect of the ScGrf3-2R Gene in Semi-Dwarf Spring Triticale (x Triticosecale Wittmack)
Source: Plants (Basel). 2022 Nov 9;11(22):3032. doi: 10.3390/plants11223032 (PMC9695017; doi:10.3390/plants11223032)
Supplement: Supplementary file 1 [file plants-11-03032-s001.zip › plants-1965769-supplementary.pdf]

Table S1. Mean values of plant traits measured in spring triticale recombinant lines derived from cross Valentin90 x Dublet based on 2018-2019 field experiment, one-way analysis of variance and Tukey's test.

**Genotype**	plant height, cm		main spike length, cm		number of spikelets, pcs	
	**2018**	**2019**	**2018**	**2019**	**2018**	**2019**
dd	91.0±1.61a	92.9±1.15a	8.78±0.10a	8.80±0.11a	22.4±0.26a	22.5±0.25a
DD	62.7±0.92b	65.3±0.96b	8.84±0.09a	8.62±0.11b	23.4±0.21b	22.1±0.26b
**Genotype**	**2018**	**2019**	**2018**	**2019**	**2018**	**2019**
SbSb	79.7±1.79a	80.8±1.82a	8.80±0.10a	8.60±0.11a	22.5±0.27a	21.9±0.26a
SaSa	71.7±1.83b	77.1±1.35b	8.78±0.10a	8.82±0.11b	23.2±0.24b	22.6±0.25b
**Genotype**	peduncle length, cm		2nd upper internode length, cm		3d upper internode length, cm	
	**2018**	**2019**	**2018**	**2019**	**2018**	**2019**
dd	31.4±0.74a	34.2±0.72a	22.5±0.40a	22.0±0.32a	12.9±0.28a	13.5±0.22a
DD	19.1±0.39b	23.8±0.51b	14.8±0.29b	14.9±0.30b	8.80±0.18b	8.85±0.19b
**Genotype**	**2018**	**2019**	**2018**	**2019**	**2018**	**2019**
SbSb	26.4±0.81a	30.2±0.87a	19.1±0.46a	18.9±0.49a	11.2±0.32a	11.2±0.31a
SaSa	22.8±0.81b	27.9±0.66b	17.5±0.52b	18.1±0.39b	10.3±0.30b	11.2±0.28a
**Genotype**	4th upper internode length, cm		2nd lower internode length, cm		1st lower internode length, cm	
	**2018**	**2019**	**2018**	**2019**	**2018**	**2019**
dd	12.1±0.39a	10.9±0.34a	8.5±0.21a	9.0±0.16a	4.0±0.17a	4.9±0.15a
DD	7.10±0.19b	6.9±0.26b	5.4±0.12b	6.0±0.15b	2.7±0.11b	3.3±0.11b
**Genotype**	**2018**	**2019**	**2018**	**2019**	**2018**	**2019**
SbSb	9.4±0.40a	9.1±0.48a	7.2±0.23a	7.6±0.24a	3.5±0.17a	4.2±0.16a
SaSa	8.3±0.41b	8.7±0.53a	6.4±0.21b	7.4±0.18a	3.1±0.14b	3.9±0.13b
**Genotype**	grain weight in main spike, g		grain number per main spike, pcs.		thousand grain weight, g	
	**2018**	**2019**	**2018**	**2019**	**2018**	**2019**
dd	1.98±0.07a	2.72±0.10a	48.9±1.40a	52.0±1.54a	39.4±0.78a	52.7±0.74a
DD	1.83±0.06b	2.57±0.08b	50.6±1.27a	52.5±1.36a	35.4±0.66b	48.9±0.75b
**Genotype**	**2018**	**2019**	**2018**	**2019**	**2018**	**2019**
SbSb	1.79±0.07a	2.48±0.09a	47.9±1.45a	49.8±1.43a	36.5±0.80a	50.1±0.80a
SaSa	1.96±0.07b	2.78±0.09b	51.3±1.36b	54.3±1.43b	37.3±0.75a	51.2±0.73b
**Genotype**	weight of the main spike, g		weight of the plant, g		harvesting index	
	**2018**	**2019**	**2018**	**2019**	**2018**	**2019**
dd	2.64±0.09a	3.44±0.11a	1.16±0.05a	1.33±0.04a	0.51±0.01a	0.56±0.01a
DD	2.51±0.07b	3.32±0.09a	0.78±0.03b	0.93±0.03b	0.54±0.01b	0.60±0.01b
**Genotype**	**2018**	**2019**	**2018**	**2019**	**2018**	**2019**
SbSb	2.44±0.08a	3.19±0.10a	0.98±0.04a	1.14±0.04a	0.51±0.01a	0.57±0.01a
SaSa	2.67±0.09b	3.54±0.10b	0.93±0.04a	1.11±0.03a	0.54±0.01b	0.59±0.01b

Table S1 (continuation)

Genotype	number of internodes, pcs.		days from sowing to heading, pcs.		days from sowing to flowering, pcs.	
	**2018**	**2019**	**2018**	**2019**	**2018**	**2019**
dd	5.20±0.05a	5.09±0.04a	58.0±0.44a	56.6±0.46a	60.9±0.48a	60.4±0.44a
DD	5.41±0.05b	5.05±0.04a	61.7±0.39a	60.9±0.56b	64.8±0.42a	62.9±0.55b
**Genotype**	**2018**	**2019**	**2018**	**2019**	**2018**	**2019**
SbSb	5.35±0.06a	5.09±0.05a	56.27±0.40a	59.4±0.70a	59.1±0.41a	62.3±0.61a
SaSa	5.28±0.05a	5.15±0.03b	57.27±0.46b	58.1±0.38b	60.3±0.49b	60.6±0.37b
**Genotype**	spike compactness		secondary shoot grain weight, g		productive tillering, spikes per plant, pcs	
	**2018**	**2019**	**2018**	**2019**	**2018**	**2019**
dd	25.6±0.24a	25.7±0.20a	2.25±0.15a	2.49±0.18a	2.39±0.08a	3.24±0.13a
DD	26.7±0.20b	25.6±0.19a	1.90±0.14b	2.57±0.32a	2.11±0.08b	3.23±0.12a
**Genotype**	**2018**	**2019**	**2018**	**2019**	**2018**	**2019**
SbSb	25.8±0.25a	25.6±0.21a	1.96±0.15a	2.40±0.19a	2.22±0.09a	3.24±0.13a
SaSa	26.6±0.21b	25.7±0.17a	2.17±0.16a	2.53±0.16a	1.98±0.06b	3.20±0.11a

Mean values ± confidence intervals are shown at a significance level of 0.05.
Mean values denoted by the same letters do not have significant differences in each group of two genotypes (*dd* and *DD*; *SbSb* and *SaSa*) calculated using one-way analysis of variance and Tukey's test, where *d-ddw1, D-Ddw1, Sb-ScGrf3- 2Rb, Sa-ScGrf3-2Ra.*

Table S2. Mean values of plant traits measured in spring triticale recombinant lines derived from cross Valentin90 x Dublet based on 2018-2019 field experiment, two-way analysis of variance and Tukey's test.

Genotype	plant height, cm		main spike length, cm		number of spikelets, pcs	
	**2018**	**2019**	**2018**	**2019**	**2018**	**2019**
ddSbSb	91.38±2.38a	96.77±1.88a	8.80±0.14a	8.81±0.14a	21.7±0.38b	22.34±0.32a
ddSaSa	92.86±2.82a	89.99±1.32b	8.90±0.20a	8.81±0.16a	23.5±0.39a	22.61±0.36a
DDSbSb	65.88±1.42b	66.14±1.55c	8.73±0.16a	8.43±0.15b	23.5±0.34a	21.51±0.38b
DDSaSa	59.78±1.24c	64.37±1.18c	8.86±0.12a	8.84±0.14a	23.4±0.29a	22.60±0.34a
**Genotype**	peduncle length, cm		2nd upper internode length, cm		3d upper internode length, cm	
	**2018**	**2019**	**2018**	**2019**	**2018**	**2019**
ddSbSb	31.65±1.09a	36.29±1.19a	22.00±0.55b	22.65±0.48a	12.93±0.43a	13.58±0.31a
ddSaSa	32.04±1.39a	32.72±0.84b	23.92±0.73a	21.59±0.42b	13.28±0.47a	13.52±0.30a
DDSbSb	20.36±0.57b	24.63±0.77c	15.78±0.49c	15.27±0.51c	9.11±0.31b	8.84±0.30b
DDSaSa	18.32±0.55c	23.18±0.67c	14.05±0.35d	14.68±0.35c	8.46±0.25b	8.86±0.25b
**Genotype**	4th upper internode length, cm		2nd lower internode length, cm		1st lower internode length, cm	
	**2018**	**2019**	**2018**	**2019**	**2018**	**2019**
ddSbSb	12.13±0.49a	10.84±0.39a	8.62±0.31a	9.30±0.25a	4.15±0.25a	5.14±0.23a
ddSaSa	12.50±0.67a	11.10±0.55a	8.52±0.34a	8.75±0.20b	4.00±0.28a	4.64±0.19b
DDSbSb	7.38±0.28b	7.01±0.37b	5.53±0.18b	5.93±0.23c	2.57±0.17b	3.27±0.16c
DDSaSa	6.78±0.29b	6.72±0.35b	5.24±0.18b	5.95±0.19c	2.68±0.16b	3.24±0.15c

Table S2 (continuation)

**Genotype**	grain weight in main spike, g		grain number per main spike, pcs.		thousand grain weight, g	
	**2018**	**2019**	**2018**	**2019**	**2018**	**2019**
ddSbSb	1.81±0.10b	2.53±0.13bc	44.2±1.93b	48.55±2.08c	39.02±1.14b	52.73±1.09a
ddSaSa	2.26±0.15a	2.86±0.14a	54.0±2.51a	54.89±2.15a	41.46±1.35a	52.78±1.00a
DDSbSb	1.76±0.10b	2.43±0.11c	51.2±2.07a	50.76±1.93bc	33.66±1.01d	47.68±1.06c
DDSaSa	1.86±0.09b	2.69±0.11ab	51.1±1.72a	53.78±1.87ab	35.95±0.89c	49.82±1.03b
**Genotype**	weight of the main spike, g		weight of the plant, g		harvesting index	
	**2018**	**2019**	**2018**	**2019**	**2018**	**2019**
ddSbSb	2.43±0.11b	3.26±0.15bc	1.11±0.06b	1.37±0.05a	0.50±0.02b	0.53±0.02b
ddSaSa	2.98±0.18a	3.57±0.15a	1.27±0.07a	1.29±0.05b	0.53±0.01a	0.58±0.01a
DDSbSb	2.41±0.11b	3.10±0.13c	0.82±0.04c	0.91±0.05c	0.53±0.01a	0.60±0.01a
DDSaSa	2.57±0.10b	3.49±0.13ab	0.73±0.03c	0.94±0.04c	0.55±0.01a	0.61±0.01a
**Genotype**	number of internodes, pcs.		days from sowing to heading, pcs.		days from sowing to flowering, pcs.	
	**2018**	**2019**	**2018**	**2019**	**2018**	**2019**
ddSbSb	5.20±0.08c	5.11±0.07ab	56.88±0.65b	55.68±0.84c	59.80±0.67b	60.49±0.80bc
ddSaSa	5.17±0.08c	5.14±0.04ab	55.00±0.48c	56.58±0.37c	57.55±0.57c	59.64±0.35c
DDSbSb	5.52±0.07a	5.05±0.07b	64.94±1.10a	62.30±0.88a	68.27±1.19a	64.13±0.79a
DDSaSa	5.35±0.05b	5.16±0.04a	57.15±0.59b	59.12±0.56b	59.9±0.63b	60.98±0.57b
**Genotype**	spike compactness		secondary shoot grain weight, g		productive tillering, spikes per plant, pcs	
	**2018**	**2019**	**2018**	**2019**	**2018**	**2019**
ddSbSb	24.92±0.34b	25.53±0.30a	2.06±0.20b	2.20±0.25b	2.36±0.12a	3.06±0.19b
ddSaSa	26.44±0.40a	25.74±0.24a	2.60±0.30a	2.69±0.24a	2.03±0.11b	3.34±0.17ab
DDSbSb	26.99±0.32a	25.60±0.28a	1.82±0.22b	2.54±0.28ab	2.07±0.13b	3.38±0.17a
DDSaSa	26.61±0.26a	25.65±0.25a	1.95±0.18b	2.34±0.22ab	1.92±0.08b	3.07±0.15ab

Mean values ± confidence intervals are shown at a significance level of 0.05.
Mean values denoted by the same letters do not have significant differences in each group of four genotypes (*ddSbSb; ddSaSa; DDSbSb; DDSaSa*) calculated using two-way analysis of variance and Tukey's test, where *d-ddw1, D-Ddw1, Sb-ScGrf3- 2Rb, Sa-ScGrf3-2Ra.*
